# Non‐destructive, whole‐plant phenotyping reveals dynamic changes in water use efficiency, photosynthesis, and rhizosphere acidification of sorghum accessions under osmotic stress

**DOI:** 10.1002/pld3.571

**Published:** 2024-03-07

**Authors:** Daniel N. Ginzburg, Jack A. Cox, Seung Y. Rhee

**Affiliations:** ^1^ Department of Plant Biology Carnegie Institution for Science Stanford California USA; ^2^ Present address: Department of Plant Sciences University of Cambridge Cambridge UK; ^3^ Present address: Plant Resilience Institute, Departments of Biochemistry and Molecular Biology, Plant Biology, and Plant, Soil, and Microbial Sciences Michigan State University East Lansing Michigan USA; ^4^ Present address: Water and Life Interface Institute East Lansing Michigan 48824 USA

**Keywords:** hydroponics, modular phenotyping, noninvasive whole‐plant phenotyping, osmotic stress, *Sorghum bicolor*, water use efficiency

## Abstract

Noninvasive phenotyping can quantify dynamic plant growth processes at higher temporal resolution than destructive phenotyping and can reveal phenomena that would be missed by end‐point analysis alone. Additionally, whole‐plant phenotyping can identify growth conditions that are optimal for both above‐ and below‐ground tissues. However, noninvasive, whole‐plant phenotyping approaches available today are generally expensive, complex, and non‐modular. We developed a low‐cost and versatile approach to noninvasively measure whole‐plant physiology over time by growing plants in isolated hydroponic chambers. We demonstrate the versatility of our approach by measuring whole‐plant biomass accumulation, water use, and water use efficiency every two days on unstressed and osmotically stressed sorghum accessions. We identified relationships between root zone acidification and photosynthesis on whole‐plant water use efficiency over time. Our system can be implemented using cheap, basic components, requires no specific technical expertise, and should be suitable for any non‐aquatic vascular plant species.

## 1 INTRODUCTION

Water and nutrient availability, along with the efficiency of their use by crops, are major determinants of agricultural productivity (Fageria et al., 2008; Sinclair & Rufty, 2012). However, unchecked demand for natural resources, such as land, fertilizer, and water, to support global agricultural production has led to widespread ecosystem degradation and biodiversity loss (Foley et al., 2005; Williams et al., 2020). Thus, the demand for stable agricultural yields amid globally changing climate and decreasing natural resource availability necessitates more resource‐efficient and climate‐resilient crops and agronomic practices.

Plant growth is neither linear over time (Paine et al., 2012) nor uniform across genotypes within a species (Mortlock & Hammer, 2000; Sugiyama et al., 2013). Measurements taken at single time points thus fail to capture the dynamic variation of plant growth across developmental stages and environmental conditions (Granier & Vile, 2014). In addition to being generally higher throughput than destructive measurements, noninvasive phenotyping approaches provide a window into temporal dynamics along a developmental trajectory and in response to changing environmental conditions. Immense progress has been made in high‐throughput phenotyping to capture plant traits noninvasively, particularly through the use of advanced imaging platforms (Li et al., 2020; Shakoor et al., 2017). For example, by noninvasively tracking growth over time in various *Setaria* spp., in which visual leaf area strongly correlates with above‐ground biomass, Fahlgren and colleagues uncovered species‐specific growth dynamics across time and environmental conditions that would have otherwise been missed by endpoint measurements alone (Fahlgren et al., 2015).

In contrast to phenotyping approaches that capture data from only certain plant parts, whole‐plant phenotyping can identify phenotypes and growth conditions that are optimal for both above‐ and below‐ground tissues and is thus likely more valuable for breeding more resilient and resource‐efficient crops (Chochois et al., 2015). Because of the relative difficulty and time required to measure traits at the whole‐plant level (Li et al., 2020), measurements are often simplified to include only above‐ground plant tissue. However, approaches that exclude root biomass are potentially limited in value. For example, identifying genetic variation in traits such as biomass accumulation and water use efficiency (WUE) is particularly important in the context of water‐limited conditions in which the ratio of root:shoot biomass is often substantially different than under water‐replete conditions (Benjamin et al., 2014; Hubick et al., 1986; Xu et al., 2015). Even in well‐watered conditions, the ratio of root:shoot biomass partitioning can be different among genotypes (Chenu et al., 2018; Chochois et al., 2015), further highlighting the importance of including below‐ground biomass to accurately quantify resource use efficiency (Leakey et al., 2019).

Various approaches can phenotype below‐ground traits noninvasively. For example, root growth can be measured in vivo using magnetic resonance imaging (MRI) (Rascher et al., 2011), X‐ray computed tomography (CT) scanning (Zeng et al., 2021), or with luminescence‐based reporters (Rellán‐Álvarez et al., 2015). When combined with non‐destructive techniques to measure above‐ground tissues, such approaches could in theory measure whole‐plant growth over time. However, these approaches often require large upfront costs, state‐of‐the‐art technologies, or specific technical expertise (Großkinsky et al., 2015). There are reports of cheaper and simpler approaches to measure whole‐plant traits non‐destructively (Fletcher et al., 2018). However, the authors found that data obtained non‐destructively did not correlate well with values from plants harvested for end‐point measurements.

To address the various limitations of measuring whole‐plant traits non‐destructively, we developed a low‐cost and versatile approach that can noninvasively measure whole‐plant physiology over time. As a proof of concept, we measured biomass accumulation and water use over time to directly quantify whole‐plant WUE. Plant WUE, broadly defined as the ratio of biomass accumulation to water use via transpiration (Vadez et al., 2014), represents an important metric to optimize for improving plant resilience and agricultural sustainability. Various methods exist to quantify WUE, ranging from instantaneous measurements at the leaf scale (Vadez et al., 2014) to long‐term quantification at the canopy scale (Lambers & Oliveira, 2019). Because of the difficulty in measuring whole‐plant WUE, it is common to extrapolate these data from leaf‐level measurements or via proxies. For example, quantifying leaf gas exchange rates can provide a measure of both photosynthetic and transpiration rates. However, instantaneous leaf‐level WUE data are often in disagreement with whole‐plant measurements at longer timescales (Medrano et al., 2015). Leaf carbon isotope discrimination (CID) is often used to estimate whole‐plant WUE as CID is strongly correlated with WUE in C_3_ species (Farquhar et al., 1982). However, the strength of this correlation is greatly reduced in C_4_ species, including important crops such as maize and sorghum (Henderson et al., 1998; Vadez et al., 2014). Moreover, quantifying CID is relatively slow and expensive (Vadez et al., 2014), thus necessitating simpler and more widely applicable methods.

In contrast to these methods, our approach, which uses hydroponic cultivation, allows for the direct and non‐destructive measurement of whole‐plant WUE through direct quantification of biomass accumulation and water use. We show that hydroponic cultivation results in WUE equal to that of soil‐grown plants and then demonstrate the temporal and contextual versatility of our approach by measuring whole‐plant WUE every two days on unstressed and osmotically stressed seedlings. In doing so, we identified relationships between root zone acidification and aspects of photosynthesis on whole‐plant growth and WUE over time. Our approach can be implemented using cheap, basic components and requires no specific technical expertise. It can be implemented for any non‐aquatic, vascular plant species and can therefore contribute broadly to identifying and engineering superior crop germplasm.

## 2 RESULTS

**FIGURE 1:**
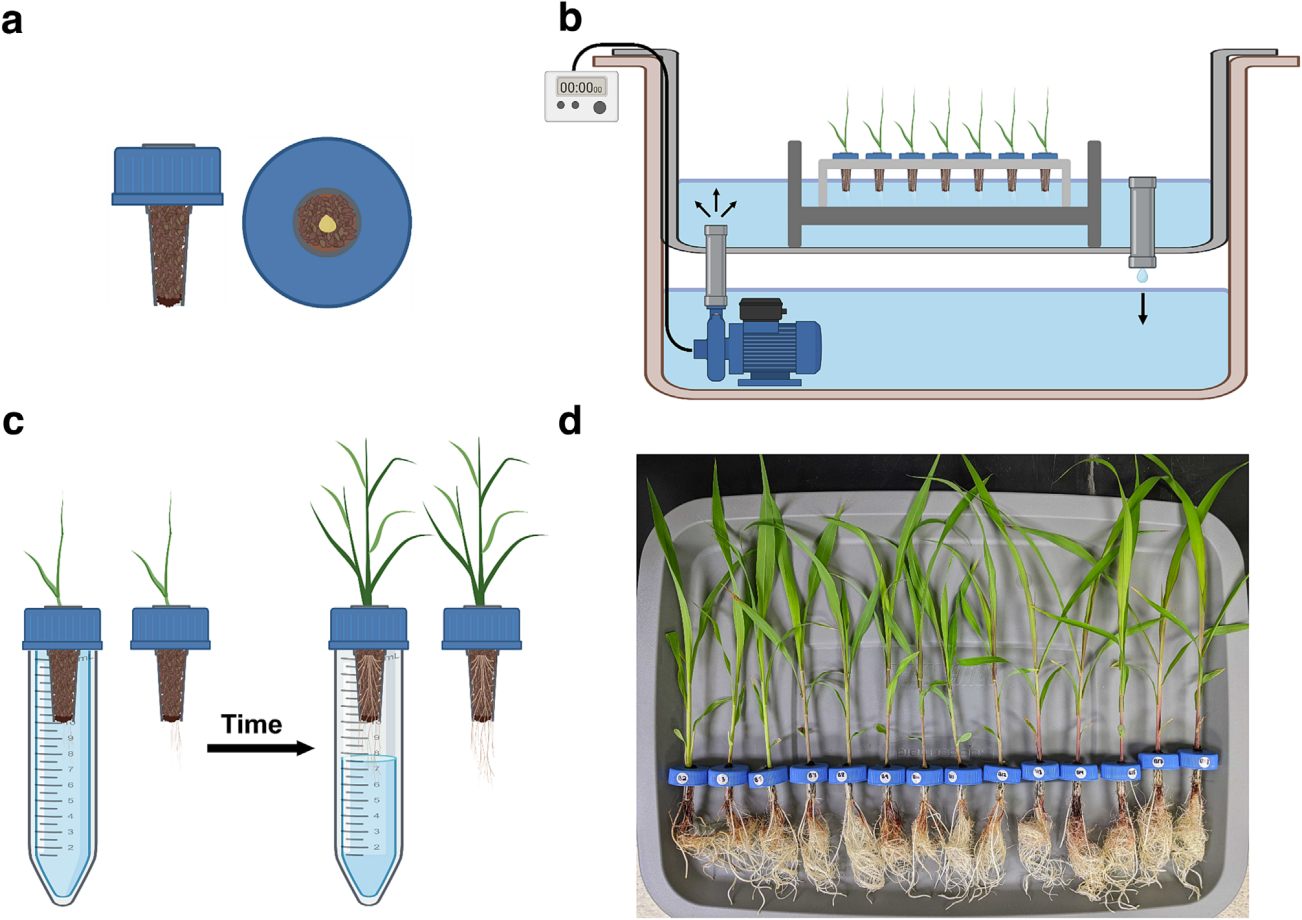
(a) Representation of a screw‐on cap with a hole drilled into the top and a cut‐off pipette tip inserted into the hole. Soil is inserted into the pipette tips and seeds are sown into the soil‐filled tips. (b) Schematic of the recirculating ebb and flow hydroponic system used in this study. Water is pumped up from the bottom reservoir up to the top reservoir, where it rises up to the point of the drain pipe and then falls back down into the bottom reservoir. Plants are placed in the top reservoir such that the water level rises up to the soil‐filled tips. The duration and time of day of irrigation events are controlled via a programmable timer connected to the pump. Samples are grown in these “open” hydroponic conditions until they reach a predetermined age or developmental stage. (c) Samples are then screwed onto tubes filled with nutrient solution, at which point the system is “closed.” When closed, water loss due to evaporation is prevented. After a designated amount of time, caps are unscrewed and the plants are lifted out of the tube. Tubes and caps with plants can be weighed to determine water use and biomass accumulation relative to previous timepoints, respectively. Caps with plants can then be screwed back onto tubes for continued growth in “closed” conditions. (d) Sorghum samples after 8 days of growth in tubes. Graphics for cartoon schematics in a–c are from BioRender.

**FIGURE 2:**
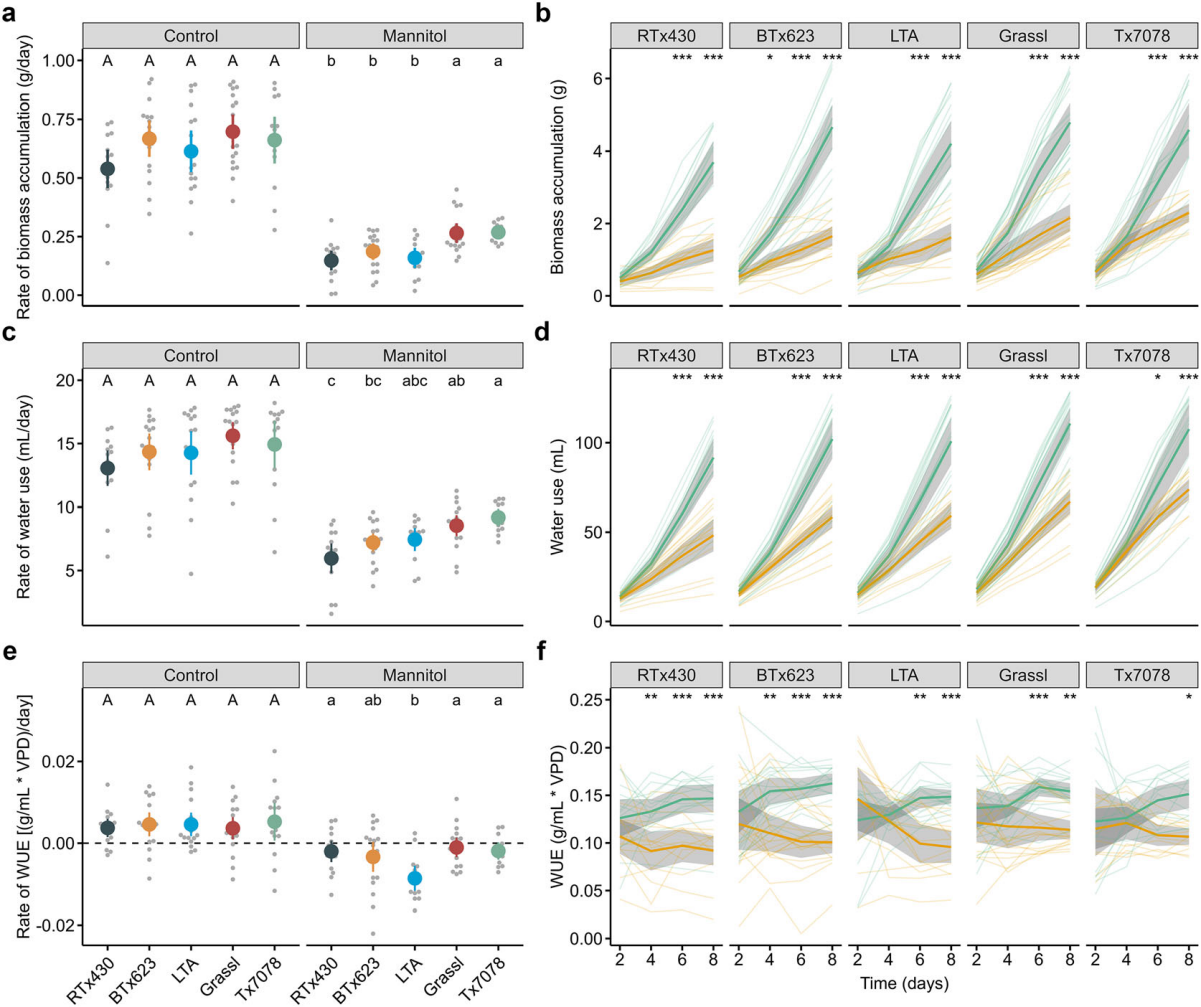
Rate of biomass accumulation (a), water use (c), and WUE (e), as determined by the slope of linear regression (*n* = 12–18 per genotype, condition, and day; *N* = 4). Letters represent statistically different genotypes as determined by 1‐way ANOVA (performed separately for control and mannitol treatments) followed by Tukey's HSD test (alpha = 0.05). Cumulative biomass accumulation (b), water use (d), and WUE (f) of all genotypes over time in control (green) or mannitol‐treated (orange) conditions. Thick solid lines in b, d, and f represent the average value per condition bounded by the 95% confidence interval. Thin lines in b, d, and f represent individual samples. Asterisks indicate statistical significance at a given time point between mannitol and control samples as determined by 2‐way ANOVA: * (*p* < 0.05), ** (*p* < 0.01), *** (*p* < 0.001). *n*, number of biological replicates per genotype and condition; *N*, number of independent experiments.

We developed a simple, cheap, and modular system to grow and phenotype whole‐plant traits non‐destructively over time. A detailed, step‐by‐step protocol can be found online (protocols.io; dx.doi.org/10.17504/protocols.io.q26g7p6m1gwz/v1). Briefly, a hole is drilled into screw‐on caps and a cut‐off pipette tip of approximately equal diameter is inserted into the hole of the screw‐on cap (Figure 1a). Soil is then inserted into the snugly‐fit pipette tips, and seeds are sown into the soil‐filled tips. Plants are initially grown in “open” hydroponic conditions such that all samples are grown in a single reservoir whose water is exposed to the air (Figure 1b). Upon reaching a desired size, age, or developmental stage, tops are screwed onto tubes and the system becomes “closed” such that water loss from the tube is restricted to uptake by the plant (Figure 1c). By unscrewing the tops, the entire plant can be removed, weighed, and then screwed back onto the tube for continued growth (Figure 1c–d). When grown this way, whole‐plant biomass accumulation and water use can be assayed repeatedly over time (Figure 2a–d). Moreover, the modularity of our approach allows for precise control of the root environment to investigate a variety of phenotypes, including the relationship of whole‐plant biomass accumulation and water use in response to changing abiotic or biotic conditions.

As a proof‐of‐concept of this approach, we sought to compare genotype‐specific variation in biomass accumulation, water use, and whole‐plant WUE of various sorghum accessions when grown either in soil or using our closed‐system hydroponic approach. Sorghum (*Sorghum bicolor*) is a highly productive and relatively stress‐resistant C_4_ crop (Ananda et al., 2020; Khalifa & Eltahir, 2023). It is among the world's most important cereal crops and is utilized for both food and bioenergy (Ananda et al., 2020; Khalifa & Eltahir, 2023). Genotype‐specific variation exists in whole‐plant WUE in soil‐grown sorghum (Balota et al., 2008; Xin et al., 2008). We selected five sorghum genotypes (Table 1) to represent a diverse range of geographic origins, taxonomic races, variation in transpiration rates measured at the leaf level (Balota et al., 2008), and variation in WUE measured at the whole‐plant level (Balota et al., 2008; Xin et al., 2008).

**TABLE 1:** Description of sorghum accessions used in this study.

Accession^a^	Race	Origin^b^	References
RTx430 (PI 629034)	Caudatum	USA	Miller, 1984; Menz et al., 2004
BTx623 (PI 564163)	Kafir × Caudatum	USA	Menz et al., 2004; Luo et al., 2016
Liang Tang Ai (PI 656046)	Bicolor	China	Xin et al., 2008
Grassl (PI 154844)	Caudatum	Uganda	Boatwright et al., 2021
Tx7078 (PI 656487)	Kafir	USA	Menz et al., 2004; Xin et al., 2008

^a^ Plant Introduction (PI) numbers from USDA's Office of Seed and Plant Introduction are included underneath accession names.

^b^ Origin refers to where the accession was bred. However, parental lines may have different origins.

We first asked whether our method of quantifying WUE is consistent with established methods (Cernusak et al., 2007; Xin et al., 2009) by comparing the WUE of plants grown in our hydroponic system to plants grown in soil. Because most traditional soil‐grown approaches to directly measure WUE are destructive, we compared end‐point biomass accumulation and water use over the course of 8 days after reaching the third leaf stage in either hydroponic or soil‐growth conditions. All genotypes accumulated more biomass and consumed more water when grown in soil compared with when grown hydroponically (Figure S2A–B). However, WUE after 8 days was indistinguishable between soil and hydroponic conditions for all genotypes (Figure S2C).

Next, we asked whether evaporative loss of water over time was negligible compared to plant water use, which is required to accurately infer plant water use from changes in tube weight over time. Water loss over time due to evaporation was measured in tubes from which whole plants were removed by cutting off roots and shoots to eliminate transpiration. Over the course of 7 days, plant‐less samples lost an average of 0.6 mL of water because of evaporation (Figure S3), equivalent to approximately 0.7% of average water use in plants grown in control conditions over the same period. Evaporative water loss in our hydroponic system was therefore determined to be negligible compared with transpirational water uptake.

Having validated our system, we next sought to compare the effect of osmotic stress on cumulative biomass accumulation, water use, and WUE across five sorghum accessions (Table 1). Specifically, we asked how these traits vary over time for each accession and whether genotype‐specific differences in plant physiology manifest in unstressed or osmotically stressed conditions. Plants were grown in tubes containing either nutrient solution alone or nutrient solution with 10 mM mannitol to induce osmotic stress. Osmotic stress resulted in decreased biomass accumulation, water use, and WUE in all genotypes, compared with control conditions (Figure 2). There were no genotype‐specific differences in the rates of biomass accumulation, water use, or WUE under control conditions (Figure 2a,c,e). However, genotype‐specific differences were observed in both the time and extent to which mannitol stress impacted growth and WUE. For example, Grassl and Tx7078 accumulated biomass at a greater rate than the other genotypes when grown in mannitol (Figure 2a). Additionally, change in biomass accumulation in mannitol conditions relative to control was observed slightly earlier in BTx623 compared to all other genotypes (Figure 2b). Despite this slight temporal difference, the overall rate of biomass accumulation in BTx623 under osmotic stress was similar to that of osmotically‐stressed RTx430 and LTA (Figure 2a). Mannitol stress resulted in the greatest reduction in water use rates in RTx430 and the smallest decrease in Tx7078 (Figure 2c). However, there were no genotype‐specific temporal differences in average water use rates under osmotic stress compared to controls (Figure 2d). With regard to WUE rates, stress‐induced reductions were most pronounced in LTA (Figure 2e). This greater reduction in WUE was independent from the time at which WUE of osmotically stressed plants dropped below control levels. For example, WUE of RTx430 and BTx623 plants grown in mannitol was decreased compared to control by Day 4, whereas osmotic stress did not reduce Tx7078 WUE until Day 8, later than all other genotypes (Figure 2f). Collectively, these results suggest that the onset of osmotic stress does not always mirror its magnitude on plant physiology over time. Additionally, these results indicate that Grassl and Tx7078 are more tolerant to osmotic stress than RTx430 and that LTA is less efficient in using water compared with other accessions when osmotically stressed at the third leaf stage.

To better understand how osmotic stress affects the relationship between biomass accumulation and water use, and to identify which underlying component is a stronger predictor of changes in WUE, we regressed biomass accumulation by water use (Figure S4A), WUE by biomass accumulation (Figure S4B), and WUE by water use (Figure S4C). There was a strong positive correlation between biomass accumulation and water use for all genotypes in both control and mannitol‐treated samples, though this relationship was stronger in control conditions (Figure S4A). A positive correlation was also found between WUE and biomass accumulation for all genotypes under both control and osmotically stressed conditions. However, the correlation was stronger for all genotypes when exposed to osmotic stress (Figure S4B). Conversely, WUE could only be explained in part by water use in certain genotypes and under certain conditions (Figure S4C). In LTA, for example, WUE increased linearly with water use only under osmotic stress, whereas there was no relationship between WUE and water use in either condition for Tx7078 (Figure S4C). For all genotypes in which a positive correlation was found between both biomass accumulation and water use with WUE, biomass accumulation was the stronger predictor for increases in WUE (Figure S4B–C). These results suggest that mannitol‐induced osmotic stress reduces WUE and that in both stressed and unstressed conditions, biomass accumulation is a stronger determinant of WUE than water use.

**FIGURE 3:**
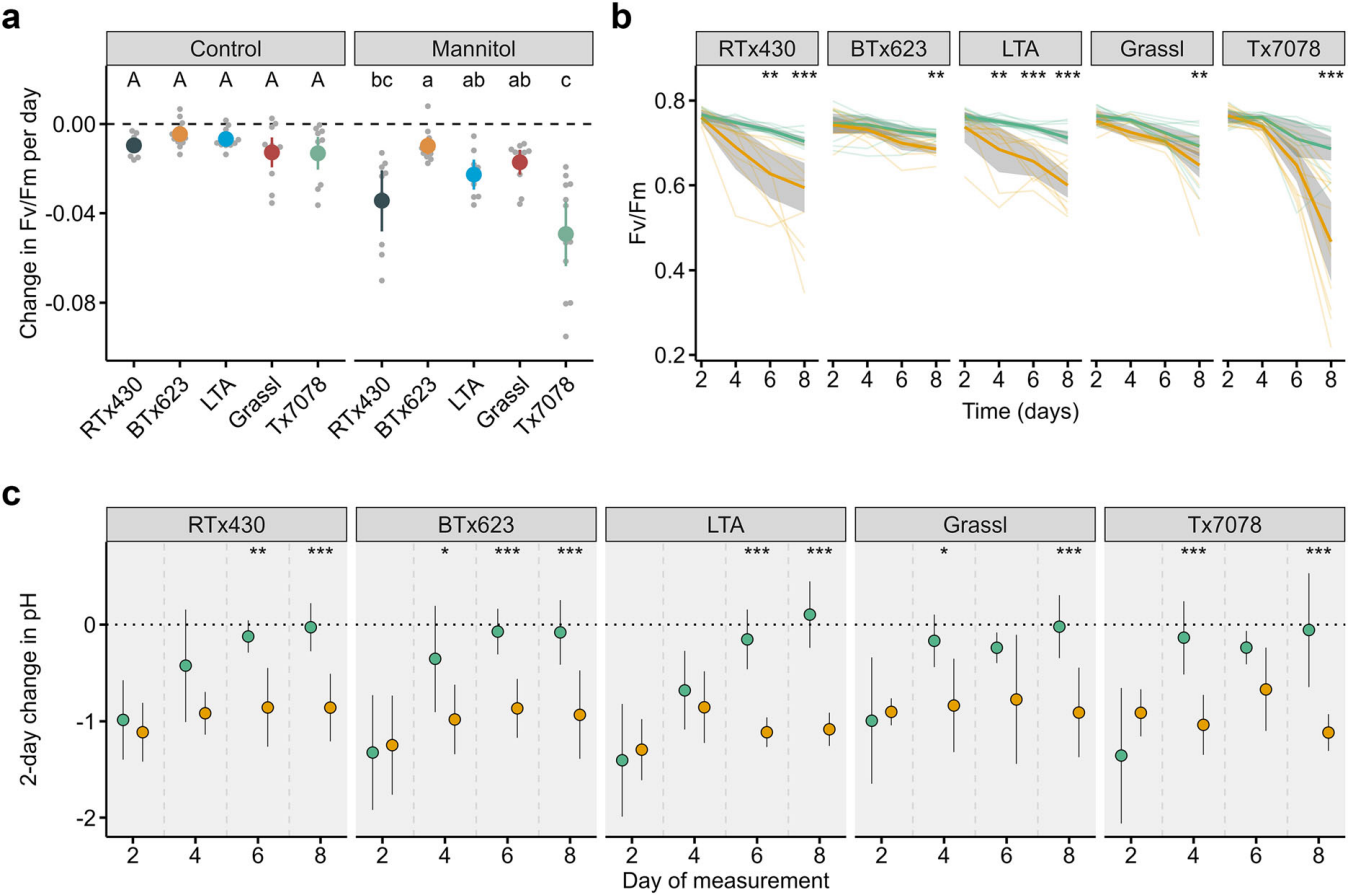
(a) Change in *F*_*V*_/*F*_*M*_ as determined by the slope of linear regression over time (*n* = 9–18 per genotype, condition, and day; *N* = 3–4). Letters represent statistically different genotypes as determined by 1‐way ANOVA (performed separately for control and mannitol treatments) followed by Tukey's HSD test (alpha = 0.05). (b) *F*_*V*_/*F*_*M*_ over time of the third true leaf of plants grown in control (green) or mannitol (orange) conditions. Thick solid lines represent the mean value per condition bounded by the 95% confidence interval. Thin lines represent individual samples. (c) Average 2‐day change in pH in the growth media in control (green) or mannitol (orange) conditions (*n* = 7–18 per genotype, condition, and day; *N* = 3–4). Note that any solution remaining in the tubes after every 2‐day period was discarded and replaced by fresh nutrient solution to ensure sufficient water for plant growth for the subsequent two days. Error bars represent standard deviation. Asterisks in b and c indicate statistical significance at a given time point between mannitol and control samples as determined by 2‐way ANOVA: * (*p* < 0.05), ** (*p* < 0.01), *** (*p* < 0.001). *n*, number of biological replicates per genotype and condition; *N*, number of independent experiments.

Having observed genotype‐specific differences in biomass accumulation, water use, and WUE rates specifically in osmotically‐stressed conditions, we next sought to identify possible mechanisms for this context‐specific difference in plant growth and physiology. Osmotic stress can impair photosynthetic performance (Chen et al., 2011; Grzesiak et al., 2006). We therefore asked whether genotype‐specific differences in biomass accumulation, water use, and WUE under mannitol stress could be explained by differences in photosynthetic efficiency. Surprisingly, Photosystem II (PSII) maximum quantum efficiency (*F*_*V*_/*F*_*M*_) of the third true leaf decreased to the greatest extent in both Tx7078 and RTx430 plants in response to osmotic stress (Figure 3a). Similar to the effects of osmotic stress on biomass accumulation and water use, the time at which *F*_*V*_/*F*_*M*_ levels dropped compared to control varied by genotype (Figure 3b). Specifically, *F*_*V*_/*F*_*M*_ in mannitol‐grown LTA plants was reduced relative to control levels by Day 4, which was earlier than all other genotypes (Figure 3b) and further demonstrates that the onset of stress does not always correlate with its magnitude. These results suggest that photosynthetic capacity *Fv/Fm* at the third leaf stage was not strongly associated with plant growth or WUE under osmotic stress, as Grassl and Tx7078 plants had equal rates of biomass accumulation and WUE under osmotic stress despite differences in the effect of osmotic stress on their PSII quantum efficiency.

We next asked if there were any differences between the accessions in their root physiology in response to osmotic stress. By exuding a diverse set of compounds from their roots, plants actively modify their root zone to facilitate nutrient uptake and in response to abiotic stress, including mannitol‐induced osmotic stress (Darko et al., 2019; Falhof et al., 2016; Marschner et al., 1986). One of the ways in which plants optimize their rhizosphere properties is via soil acidification (Ehrenfeld et al., 2005; Marschner et al., 1986). Growing plants in closed tubes makes it possible to directly and easily measure rhizosphere acidification. We therefore asked whether and when there were any genotype‐specific differences in pH change of the media every 2 days (delta‐pH). The pH of media with or without mannitol was measured before adding it to tubes, and after 2 days of plant growth (Figure 3c and Data S1). Any solution remaining in the tubes after every 2 days was then discarded and fresh nutrient solution was added to ensure sufficient water for plant growth for the subsequent 2 days. After 2 days of growth in closed tubes, there was no effect of osmotic stress on delta‐pH in any genotype ; both control and mannitol‐supplemented media were acidified by 1.0‐1.5 in all genotypes (Figure 3c). However, control plants stopped acidifying their media within 4 to 6 days, whereas the mannitol‐supplemented media was consistently acidified throughout the experiment by plants of all genotypes (one‐sample t‐test; (alpha = 0.05), Figure 3c, and Data S2). These results suggest that prolonged root zone acidification may be an adaptive response conserved among sorghum accessions in response to osmotic stress at the seedling stage.

We have demonstrated how non‐destructive, whole‐plant phenotyping can be employed using simple and readily available components. We showcase the versatility of our approach by directly measuring multiple traits across a diverse set of sorghum accessions in unstressed and osmotically stressed conditions. Collectively, we illustrate how this method can identify genotype‐specific temporal dynamics in plant growth across multiple organs and physiological processes.

## 3 DISCUSSION

Plant phenotypes are dynamic manifestations of genetics, environmental conditions, and developmental stage. Phenotypic plasticity is evidenced by changes in growth of both above‐ and below‐ground tissues. Non‐destructive, whole‐plant phenotyping facilitates quantification of dynamic growth processes that are more challenging to identify via destructive approaches and is therefore crucial for improving our understanding of plant growth and engineering more resilient and resource‐efficient crops. However, noninvasive, whole‐plant phenotyping approaches are often expensive and technically complex. To address these limitations, we developed a cost‐effective, simple, and modular approach that can measure whole‐plant physiology over time. Unlike soil‐grown conditions, hydroponic cultivation allows us to physically isolate roots and return them to their growth environment non‐destructively. In doing so, entire plant biomass can be measured directly and repeatedly. Additionally, if each plant is grown physically separated from other plants and evaporation is prevented, plant water use can also be measured directly. These capabilities alone are of practical value for the study of plant growth and development given that these traits are not readily quantifiable in a direct, non‐destructive manner for soil‐grown plants. Moreover, quantification of these traits over time allows for direct measurements of whole‐plant WUE over time. Previous studies have demonstrated creative approaches to quantifying WUE non‐destructively (Fahlgren et al., 2015; Fletcher et al., 2018). However, these methods either ignore root biomass contributions (Fahlgren et al., 2015) or do not correlate well with WUE values from harvested soil‐grown plants (Fletcher et al., 2018).

Comparing our hydroponic system to soil‐grown plants, we observed that plant growth and resource usage were strongly influenced by abiotic conditions. This was made clear by the greater biomass accumulation and water use of soil‐grown plants compared with those grown hydroponically. Interestingly, WUE was uniform across growth conditions. This suggests that biomass accumulation at this stage of development necessitates a proportionally equal amount of water use, regardless of absolute growth rates. What might explain slower growth rates in the hydroponic system compared with potted soil? The hydroponic growth conditions used in this study were chosen to be suitable for a diverse range of genotypes at a specific developmental stage, rather than being designed to optimize growth. However, irrigation scheduling, nutrient composition, and abiotic conditions for hydroponic growth such as media oxygenation could all be refined to optimize a given metric, as desired.

We chose to examine the five varieties of sorghum (Table 1) because, previously, genotype‐specific differences in whole‐plant WUE have been observed in soil‐grown sorghum (Mortlock & Hammer, 2000; Xin et al., 2008, 2009). We specifically focused on identifying genotype‐specific variation at the seedling stage as sorghum biomass accumulation at the time of harvest is most affected by drought‐induced osmotic stress when stress occurs at the seedling stage (Mastrorilli et al., 1999), similar to the heightened sensitivity of many crop species to stresses at early stages in their development, such as maize (Ge et al., 2012; Kang et al., 2000), rice (Zhao et al., 2014), and tomato (Sivakumar et al., 2020). Under non‐stressed conditions, LTA previously exhibited greater whole‐plant WUE than Tx7078 when grown in soil, but equal WUE to BTx623 and RTx430 (Xin et al., 2008; Xin et al., 2009). That we did not observe genotype‐specific differences in WUE in either soil‐ or hydroponically grown sorghum in control conditions could reflect the different ages and developmental stages of the plants being assayed. We measured plant growth starting at the third leaf stage, corresponding to plants that were approximately 2 weeks old, whereas plants were approximately 4–6 weeks old (corresponding to the eighth leaf stage) in Xin et al. (2008) and Xin et al. (2009). The differences in WUE we observed compared with previous reports further support the idea that WUE varies by developmental stage (Fahlgren et al., 2015). We did, however, observe genotype‐specific differences in WUE when plants were grown under osmotic stress. In light of the previously identified superiority of LTA in terms of its WUE when grown in soil under non‐stressed conditions, it was somewhat surprising that LTA was most sensitive to osmotic stress in terms of its WUE. Although WUE of many species, including sorghum (Xie & Su, 2012), increases in response to drought stress in soil‐grown conditions (Peters et al., 2018), mannitol‐induced osmotic stress decreases WUE in multiple species (Cha‐Um & Kirdmanee, 2008; Darko et al., 2019), similar to what we observed with sorghum in this study. Collectively, these findings highlight the importance of considering multiple developmental stages and abiotic conditions when studying plant resilience.

Because WUE is underpinned by both biomass accumulation and water use, much work has been done to identify which of these components should be optimized to improve crop WUE. Whether WUE is even a desirable agronomic trait is actually a contentious matter (Blum, 2009; Condon et al., 2004). This is because, in many crop species, increased WUE is usually achieved through reduced stomatal conductance, which in turn results in decreased carbon fixation and lower yields (Blum, 2009; Vadez et al., 2014). Other work, however, has demonstrated that higher WUE via reduced stomatal conductance does not necessitate a yield penalty if there is a concomitant increase in photosynthetic rates (Vadez et al., 2014). These contradictory findings suggest that the contributions of growth and water use to WUE are both complex and are underpinned by both genotypic and environmental factors. In all such cases, however, it is generally agreed that increasing, rather than decreasing soil water use, is a key breeding strategy for improving both crop yield and potentially WUE (Blum, 2009; Condon, 2020). This is because increased plant water use would support more carbon capture and reduce the fraction of soil water lost to evaporation. Moreover, increased root growth could facilitate water capture from deeper in the soil profile where water is less prone to evaporative losses (Condon, 2020). These findings suggest that breeders should aim to identify genotypes that maximize water use and biomass accumulation, as these traits should support both high yields and plant resilience during water stress (Blum, 2009; Condon, 2020). This idea is supported by the findings in this study, given that WUE positively correlated with both increased biomass accumulation and water use and that these relationships were stronger under osmotic stress. Moreover, biomass accumulation, rather than water use, was a stronger predictor of WUE in all genotypes, which has previously been observed in soil‐grown sorghum (Xin et al., 2009). These results further support the idea that breeders should aim to identify the fastest growing lines to both optimize yields and WUE while bearing in mind the complex interactions of WUE with plant genetics, developmental stage, and abiotic conditions.

The ability to track plant growth over time non‐destructively can reveal biological insights that could be missed through endpoint analysis alone (Fahlgren et al., 2015). For example, average WUE of mannitol‐treated RTx430 and BTx623 plants was already lower than control plants by Day 4, yet *F*_*V*_/*F*_*M*_ in those same plants was not yet distinguishable from control plants, indicating that WUE in sorghum is negatively affected by osmotic stress before maximum quantum efficiency of Photosystem II is affected. Also, because biomass accumulation was already reduced by Day 4 in mannitol‐treated BTx623 plants compared with control plants, something other than a reduction in *Fv/Fm* was impairing growth rates in that accession. End‐point analysis would not have revealed how osmotic stress impacted growth and *Fv/Fm* differently at different times among the genotypes. Non‐destructive phenotyping also brought to light that stress response onset does not necessarily correlate with stress response magnitude, as evidenced by the late, but severe drop in *F*_*V*_/*F*_*M*_ in Tx7078 plants compared with the other genotypes. Previous work utilizing non‐destructive phenotyping techniques has also identified a disconnect between stress response onset and magnitude in Arabidopsis (Awlia et al., 2016). Collectively, these observations further support the value of non‐destructive approaches to capturing the dynamic nature of plant phenotypes across time and environments (Granier & Vile, 2014).

Plants actively modify the soil properties of their growing environment to optimize growth by improving nutrient uptake and mobilization and to regulate soil microbial dynamics (Falhof et al., 2016; Marschner et al., 1986; Shen et al., 2013). Depending on the biotic and abiotic conditions in the soil, roots can exude sugars, amino acids, enzymes, organic acids, and phytosiderophores to optimize conditions for symbiotic rhizospheric microorganisms (Ryan et al., 2009) or for direct nutrient uptake (Marschner et al., 1986; Shen et al., 2013). The non‐destructive nature of our closed‐system hydroponic approach allowed us to more readily identify a connection between root zone acidification and biomass accumulation than had we grown plants in soil for destructive end‐point analyses. For example, after 2 days of growth in closed tubes, both control and mannitol‐treated plants of all genotypes acidified their root zones equally. At this time point, there was also no difference in total biomass accumulation between control and mannitol‐treated samples in any genotype. Although root zone acidification gradually decreased over time in control samples of all genotypes, it remained fairly constant for mannitol‐treated plants. That mannitol‐treated plants continued to acidify their root zone throughout the experiment suggests a greater and prolonged investment of root exudates to actively modify their root environment. One explanation could be that mannitol stress can impair the uptake and translocation of phosphorus (Resnik, 1970) and potassium (Slama et al., 2007). Interestingly, the average pH after every 2 days of growth in mannitol was 5.5–5.8 (Data S1), which is a range that favors plant uptake of all essential nutrients (Pennisi & Thomas, 2005; Reed, 1996). Thus, root zone acidification in response to mannitol stress could be an adaptive response to improve mineral nutrient uptake and mobility.

Various aspects of the dynamic effects of root zone conditions on plant growth remain poorly understood (Kardol et al., 2013). Additionally, many plant responses to environmental conditions are age‐dependent (Bond, 2000; Panter & Jones, 2002). Thus, although hydroponic cultivation is an established approach in plant biology research (Asao, 2012), the isolation and modularity of the root zone environment afforded by our approach open the door to additional avenues of experimentation for directly studying dynamic and age‐dependent processes. For example, researchers could finely control the characteristics of the solution in each tube to investigate the temporal effects of mineral nutrition or biotic stress on whole‐plant development and physiology and to analyze how these characteristics differentially affect shoot versus root growth. Our approach also makes it easier to investigate how changes in root architecture and morphology over time influence plant growth. Although currently our approach does not distinguish shoot from root biomass, it could easily be paired with imaging‐based phenotyping technologies such as PhenoBox (Czedik‐Eysenberg et al., 2018) or PlantCV (Gehan et al., 2017) to quantify both components separately. Future studies that incorporate such imaging technologies could directly quantify the relationship between root zone acidification and root growth characteristics.

Another future application would be to examine the effect of root zone oxygenation on growth. Some plant species require more oxygen in their root zone than others (Asao, 2012; Narsai et al., 2011). Supplementing hydroponic nutrient solution with hydrogen peroxide can increase the availability of dissolved oxygen in the media and can increase nutrient uptake and chlorophyll content in various species (Butcher et al., 2017; Liu et al., 2022). Although suitable concentrations of hydrogen peroxide would need to be established to avoid phytotoxicity, its use in the nutrient solution would not require any physical modifications to the closed‐hydroponic design. Additionally, tubes could be opened more frequently than every 2 days to further increase root zone oxygenation. Although 50‐mL tubes were sufficiently large for sorghum plants at the third leaf stage, larger tubes could be used for either older or larger species, as required.

Efforts to identify and engineer more resource‐efficient and resilient crops will benefit from phenotyping approaches that can noninvasively quantify whole‐plant growth and physiology over time. Our approach allows for inherently greater throughput than traditional approaches and can, in its entirety, be implemented using cheap and readily available components. Its versatility for studying various aspects of plant growth and physiology makes it a valuable method for plant scientists in studying plant growth as a whole and identifying superior and more resilient crops.

## 4 MATERIALS AND METHODS

A detailed, step‐by‐step protocol can be found at protocols.io; dx.doi.org/10.17504/protocols.io.q26g7p6m1gwz/v1.

An R notebook containing scripts for visualizing and analyzing data from these experiments can be found at https://rpubs.com/danny_ginzburg/1143647.

### 4.1 Hydroponic growth apparatus construction and seed planting

Screw‐on tops of 50‐mL Falcon tubes were used to physically support plant growth and to secure individual tubes to prevent evaporative water loss. To physically support growing plants, a hole was drilled into the center of a 50‐mL Falcon tube cap using a 21/64‐in. drill bit. A 1250‐μL pipette tip was cut ~3 cm from the tip (narrower end) and inserted into the pre‐drilled Falcon tube cap such that the tip fit snugly into the hole in the cap and rested almost flush with the top of the cap. Rapid‐Rooter starter plugs (General Hydroponics, Santa Rosa, CA, USA) were cut into rectangular slices of ~0.5‐cm length and ~0.9‐cm width and each slice was inserted into the top of each tip and then gently pushed to the bottom. To prevent unnatural water retention and pressure buildup in the filled tips, pinholes were created in the tips ~25% from the bottom using a needle. Caps with tips were then filled with fresh Pro Mix Bio‐Fungicide potting soil (Premier Tech Horticulture, Quakertown, PA, USA), placed in tube racks, and left to soak overnight in tap water, which reached the bottom of the tops but did not submerse the caps. The next day, individual sorghum seeds were planted into soil‐filled tips. While still in racks, all samples were then placed into an in‐house built ebb and flow (flood & drain) hydroponic system to germinate the seeds and support root emergence.

Briefly, an ebb and flow system utilizes a submersible pump to recirculate nutrient‐rich water between two or more reservoirs. Plants are placed in the top reservoir and grown hydroponically. The ebb and flow system used in this study consisted of two, 18‐gal Rubbermaid Roughneck plastic totes (reservoirs) (Rubbermaid, Atlanta, GA, USA), one stacked on top of the other (Figure S1A). Two, ¾‐in. diameter holes were drilled in opposite corners of the top reservoir. A ¾‐in. threaded and barbed bulkhead fitting (Figure S1B) (Botanicare, Vancouver, WA, USA) was inserted from above into one of the drilled holes and fastened from below with a rubber washer. Water in an ebb and flow system needs to be pumped up to, but not submerge, the seedlings in the upper reservoir. Thus, to increase the irrigation height, the water reaches in the upper reservoir before draining back down, a threaded height extender (Figure S1B) was then screwed into this first hole from above. The second hole that was drilled into the upper reservoir was then fitted with a threaded, plastic debris screen (Figure S1B) to allow water to gently pump up into the top reservoir. In the bottom reservoir sat an 8 W, 800‐L/h submersible pump (VIVOSUN, Ontario, CA, USA) connected via PVC plastic tubing to the barbed end of the bulkhead fitting (Figure S1A). When turned on, water is pumped up to the top reservoir from below and rises up to the level of extender before draining back down to the bottom reservoir. In order for water to constantly recirculate during irrigation, sufficient water must be added to the ebb and flow system to allow for filling the top reservoir up to the draining height and to ensure enough water is in the bottom reservoir to be pumped back up. To control the duration and number of irrigation events each day, the pump was connected to a digital programmable timer (BN‐LINK, Santa Fe Springs, CA, USA).

### 4.2 Seed germination and root emergence in hydroponic growth conditions

The drainage height of the ebb and flow system was optimized such that, during irrigation, the bottom ~2–3 cm of the cut pipette tips would be submerged in water (Figure 1b). Before seedling emergence, samples were irrigated thrice daily with tap water for 5 min every 8 h using a programmable timer as described above. Upon root emergence (root tips growing below the bottom of the pipette tip) of 50% of the samples, seedlings were irrigated every hour with a single, 15‐min irrigation event using a programmable timer. At this point, Peters Professional General Purpose 20‐20‐20 soluble fertilizer (ICL Specialty Fertilizers, Tel Aviv, Israel) was directly added to the lower water reservoir, using a 50‐mL plastic scoop, up to an electrical conductivity (EC) of 1200 mS/cm (~1.25‐g fertilizer/L water) while the water was being circulated. EC was measured with a Hanna Edge conductivity meter (Hanna Instruments, Rhode Island, USA). Upon root emergence of all samples, irrigation duration was increased to 45‐min events every hour followed by 15 min of no irrigation to increase root zone oxygenation. The reservoir was emptied and refilled with fresh nutrient solution every 3–4 days. All experiments were conducted in a greenhouse with ~600 μmol m^−^^2^ s^−^^1^ PPFD supplemental lighting from high‐pressure sodium lamps from 7 am–7 pm daily.

### 4.3 Transitioning samples from open to closed hydroponic conditions

Once 50% of plants reached the third true‐leaf stage, a bulk nutrient solution was prepared such that 40 mL of solution could be allocated to each plant in 50‐mL Falcon tubes. Nutrient solution in 50‐mL tubes consisted of 0.4 g/L Hoagland's No. 2 basal salt mixture (Sigma Aldrich Chemical Co. St. Louis, MO, USA) supplemented with ~0.5 g/L Professional General Purpose 20‐20‐20 fertilizer up to an EC of 1200 mS/cm. This bulk nutrient solution was then split into two, and mannitol was added into one of the solutions to a concentration of 10 mM. pH of both control and mannitol‐supplemented solutions was then recorded. Fifty mL tubes were then filled with 40 mL of either control or mannitol‐supplemented nutrient solution. Tubes were wrapped in aluminum foil to prevent algal growth. Tubes with solution, but no caps, were then weighed. Caps containing seedlings were then brought into the lab and were firmly screwed onto individual tubes. Tubes with seedlings screwed on were then weighed to determine initial seedling weight. If the roots of any sample did not reach the nutrient solution when fully closed into the cap, additional solution was added and the tube (with and without cap) was reweighed. Samples were evenly spaced into 50‐mL tube racks and then returned to the greenhouse.

### 4.4 Quantifying biomass accumulation, water use, and change in media pH

Every 2 days, fresh nutrient solution was prepared and pH was measured as described above for both control and 10 mM‐treated samples. Seedlings and caps were unscrewed from their tubes and placed back into the ebb and flow system (operating with constant irrigation) to keep the roots hydrated while tubes were weighed. Fifty mL tube caps (without holes) were screwed onto each tube to prevent evaporation. Tubes were brought back into the lab and were individually weighed (without caps). pH of the solution in each tube was also recorded at this time. Tubes were emptied of their solution and refilled with new control or 10 mM mannitol‐supplemented solution up to 40 mL as described above. Tubes without caps were then weighed again and caps were screwed back on to prevent evaporation. Seedlings were brought into the lab, individually weighed, and then screwed back onto their corresponding tubes before being returned to the greenhouse.

### 4.5 Chlorophyll fluorescence quantification

In the greenhouse, seedlings were gently laid on their side and dark adaptation clips were placed onto the middle of the third true leaf of each sample for 30 min. After dark adaptation, *F*_*V*_/*F*_*M*_ of the third true leaf was recorded using a chlorophyll fluorometer (OS30p+, Opti‐Sciences, Inc. Hudson, New Hampshire). Specifically, minimum fluorescence (*F*_0_) was measured after the application of a weak modulated pulse of 0.1‐μmol m^−2^ s^−1^ PPFD to the sampled region. A 1 s saturating pulse of 6000‐μmol m^−2^ s^−1^ PPFD was subsequently applied to the same region to determine maximum fluorescence (*F*_*M*_). *F*_*V*_/*F*_*M*_ was calculated as (*F*_*M*_ − *F*_0_)/*F*_*M*_. *F*_*V*_/*F*_*M*_ measurements were always made between 2 and 3 pm, as chlorophyll fluorescence parameters are under circadian regulation (Dodd et al., 2014). Samples were then placed upright back into tube racks.

### 4.6 Determination of vapor pressure deficit

In addition to genetic and developmental factors, plant transpiration rates are driven by vapor pressure deficit (VPD) (Mortlock & Hammer, 2000; Vadez et al., 2014; Grossiord et al., 2020), which is the difference between ambient vapor pressure and vapor pressure in saturated air (Grossiord et al., 2020). To facilitate comparisons of WUE across time and experiments, we normalized WUE at each timepoint by the average cumulative daytime VPD (Sinclair, 2012; Vadez et al., 2014). Temperature and humidity were measured continuously with a Govee H5075 thermometer‐hygrometer (Govee Moments Trading Limited, Hong Kong) and were averaged over 15‐min intervals. VPD was derived from temperature and RH values using the following equations from Murray (1967):

1. Saturation vapor pressure (SVP) in kPA = 0.61078 * e^(*T*/(*T* + 237.3) × 17.2694)^ where *T* is temperature in degrees Celsius
2. Actual vapor pressure (AVP) = (SVP * RH)/100 where RH is percent relative humidity
3. VPD = SVP − AVP

Daytime VPD was calculated as the average VPD during the hours when supplemental lighting was provided in the greenhouse, namely, 7 am–7 pm.

### 4.7 Determination of evaporative loss in closed‐hydroponic system

Plants were grown hydroponically in soil‐filled pipette tips as described above. At the third leaf stage, roots and shoots were cut from the bottom of the tips and the base of the cap, respectively, to prevent plant water uptake. An equal number of tubes as caps were filled with 1200 EC nutrient solution and weighed, as described above. Caps from which roots and shoots were cut off were then screwed onto nutrient‐filled tubes. Samples were placed in the same greenhouse as described above. Over the course of 7 days, caps were removed from tubes and tubes were weighed to determine average daily and cumulative water loss due to evaporation. Caps were then screwed back on after weighing. This experiment was repeated three times. Average 7‐day water use on control plants was derived from the linear regression model of water use as a function of time. Cumulative water loss of plant‐less samples was then divided by the computed 7‐day plant water use to represent the percentage of plant water use.

### 4.8 Determination of water use efficiency from soil‐grown plants

Seeds were planted in pots filled with an equal amount, by weight, of Pro Mix Bio‐Fungicide potting soil. Soil‐grown plants were grown in the same greenhouse as hydroponically grown plants, as described above. When 50% of the samples reached the third true‐leaf stage, at least three samples per genotype were harvested to determine the average total biomass per genotype (initial biomass). Samples harvested at the third true‐leaf stage were visually of similar size to those that were not yet harvested. For the remaining samples, pots were watered up to the point of soil saturation and were then weighed. The soil surface and drainage holes at the bottom of each pot were then covered with aluminum foil to prevent evaporative water loss. After 8 days of growth, the aluminum foil was removed and pots with plants were weighed. Plants were then harvested to determine final, whole‐plant biomass. Eight‐day biomass accumulation was calculated as (final biomass − initial biomass). Water use was calculated as (initial pot weight − average initial biomass) − (final pot weight − final whole‐plant biomass). Soil‐grown WUE was normalized by average cumulative daytime VPD, as described above.

### 4.9 Statistical analyses

Linear regressions and their statistical comparisons were calculated using the lm() and lstrends() functions, respectively, from the ‘lsmeans’ package (Lenth, 2016) in R version 4.2.2. Differences in control versus mannitol averages at each time point were calculated by two‐way ANOVA followed by Tukey's HSD test (*p* < 0.05) using the aov() function from the Base R “stats” package (R Core Team, 2021). Slopes and *r*‐squared values from linear regressions were calculated using the stat_regline_equation() function from the “ggpubr” package (Kassambara, 2023). Comparisons of biomass accumulation, water use, and WUE between soil‐ and hydroponically grown samples were calculated by two‐way ANOVA followed by Tukey's HSD test (*p* < 0.05) using the lsmeans() function.

## AUTHOR CONTRIBUTIONS

Daniel N. Ginzburg conceived of and designed the closed‐hydroponic apparatus. Daniel N. Ginzburg and Seung Y. Rhee conceived of the experimental design. Daniel N. Ginzburg and Jack A. Cox performed the growth experiments. Jack A. Cox and Seung Y. Rhee provided intellectual contribution and input into the manuscript organization. Daniel N. Ginzburg wrote the manuscript, and Seung Y. Rhee edited the manuscript. Seung Y. Rhee advised Daniel N. Ginzburg and Jack A. Cox.

## CONFLICT OF INTEREST STATEMENT

The authors declare no conflict of interest.

## Supporting information


**Figure S1:** Images of Botanicare fittings used in the ebb and flow system. (A) threaded bulkhead fitting which is inserted into both holes that are drilled into the upper reservoir; (B) threaded plastic debris screen; (C) threaded height extender; (D) debris screen screwed onto bulkhead fitting for water inflow into the upper reservoir; (E) debris screen screwed onto two height extenders for water flowing out of the upper reservoir and back down into the lower reservoir. Images of Botanicare fittings are from https://www.hawthornegc.com.
**Figure S2:** (A) Biomass accumulation, (B) water use, and (C) WUE of plants grown in soil (brown) or hydroponically (blue). Error bars represent the standard deviation (n = 8–22; N = 3–4). Letters represent significantly different groups as determined by two‐way ANOVA followed by Tukey's HSD test (alpha = 0.05). n, number of biological replicates per genotype and condition; N, number of independent experiments.
**Figure S3:** Water loss due to evaporation in closed hydroponic tubes. Black dots represent average cumulative evaporative water loss from closed tubes from which plant stems and roots were removed. Error bars represent the standard deviation (n = 7–23; N = 2–3). n, number of biological replicates per genotype and condition; N, number of independent experiments.
**Figure S4:** (**A**) Cumulative biomass accumulation as a function of cumulative water use, (**B**) WUE as a function of total (8‐day) biomass accumulation, and **(C**) WUE as a function of total (8‐day) water use in control (green) or mannitol‐treated (orange) conditions (n = 12–18 per genotype, condition, and day; N = 4). Thick solid lines represent the treatment‐level linear regression bounded by the 95% confidence interval. Thin lines in A represent linear regressions of individual samples. β values represent the slope (coefficient) of the linear regression. r^2^ values represent the square of the Pearson correlation coefficient of the linear regression. Asterisks indicate whether the linear relationship is statistically significant: * (p‐value < 0.05), ** (p‐value < 0.01), *** (p‐value < 0.001). Absence of a regression line indicates a non‐significant linear relationship (p‐value > 0.05). n, number of biological replicates per genotype and condition; N, number of independent experiments.

**File S1:** Initial pH, final pH, and delta‐pH for all genotypes, conditions, and days of measurement.

## Data Availability

All study data are included in the main text and supporting information.

## References

[pld3571-bib-0001] Ananda, G. K. S. , Myrans, H. , Norton, S. L. , Gleadow, R. , Furtado, A. , & Henry, R. J. (2020). Wild sorghum as a promising resource for crop improvement. Frontiers in Plant Science, 11, 1108. 10.3389/fpls.2020.01108 32765575 PMC7380247

[pld3571-bib-0002] Asao, T. (Ed.). (2012). Hydroponics—A standard methodology for plant biological researches. InTech Open. 10.5772/2215

[pld3571-bib-0003] Awlia, M. , Nigro, A. , Fajkus, J. , Schmoeckel, S. M. , Negrão, S. , Santelia, D. , Trtílek, M. , Tester, M. , Julkowska, M. M. , & Panzarová, K. (2016). High‐throughput non‐destructive phenotyping of traits that contribute to salinity tolerance in Arabidopsis thaliana. Frontiers Plant Science, 7, 1414. 10.3389/fpls.2016.01414 PMC5039194 27733855

[pld3571-bib-0004] Balota, M. , Payne, W. A. , Rooney, W. , & Rosenow, D. (2008). Gas exchange and transpiration ratio in sorghum. Crop Science, 48, 2361–2371. 10.2135/cropsci2008.01.0051

[pld3571-bib-0005] Benjamin, J. G. , Nielsen, D. C. , Vigil, M. F. , Mikha, M. M. , & Calderon, F. (2014). Water deficit stress effects on corn (*Zea mays*, L.) root:shoot ratio. Open Journal of Soil Science, 4, 151–160. 10.4236/ojss.2014.44018

[pld3571-bib-0006] Blum, A. (2009). Effective use of water (EUW) and not water‐use efficiency (WUE) is the target of crop yield improvement under drought stress. Field Crops Research, 112, 119–123. 10.1016/j.fcr.2009.03.009

[pld3571-bib-0007] Boatwright, J. L. , Brenton, Z. W. , Boyles, R. E. , Sapkota, S. , Myers, M. T. , Jordan, K. E. , Dale, S. M. , Shakoor, N. , Cooper, E. A. , Morris, G. P. , & Kresovich, S. (2021). Genetic characterization of a *Sorghum bicolor* multiparent mapping population emphasizing carbon‐partitioning dynamics. G3: Genes|Genomes|Genetics, 11, jkab060. 10.1093/g3journal/jkab060 33681979 PMC8759819

[pld3571-bib-0008] Bond, B. J. (2000). Age‐related changes in photosynthesis of woody plants. Trends in Plant Science, 5, 349–353. 10.1016/S1360-1385(00)01691-5 10908880

[pld3571-bib-0009] Butcher, J. D. , Laubscher, C. P. , & Coetzee, J. C. (2017). A study of oxygenation techniques and the chlorophyll responses of *Pelargonium tomentosum* grown in deep water culture hydroponics. HortScience, 52, 952–957. 10.21273/HORTSCI11707-16

[pld3571-bib-0010] Cernusak, L. A. , Aranda, J. , Marshall, J. D. , & Winter, K. (2007). Large variation in whole‐plant water‐use efficiency among tropical tree species. New Phytologist, 173, 294–305. 10.1111/j.1469-8137.2006.01913.x 17204076

[pld3571-bib-0078] Cha‐Um, S. , & Kirdmanee, C. (2008). Effect of osmotic stress on proline accumulation, photosynthetic abilities and growth of sugarcane plantlets (Saccharum officinarum L.). Pakistan Journal of Botany, 40(6), 2541–2552.

[pld3571-bib-0012] Chen, W. , Feng, C. , Guo, W. , Shi, D. , & Yang, C. (2011). Comparative effects of osmotic‐, salt‐ and alkali stress on growth, photosynthesis, and osmotic adjustment of cotton plants. Photosynthetica, 49, 417–425.

[pld3571-bib-0013] Chenu, K. , Van Oosterom, E. J. , McLean, G. , Deifel, K. S. , Fletcher, A. , Geetika, G. , Tirfessa, A. , Mace, E. S. , Jordan, D. R. , Sulman, R. , & Hammer, G. L. (2018). Integrating modelling and phenotyping approaches to identify and screen complex traits: Transpiration efficiency in cereals. Journal of Experimental Botany, 69, 3181–3194. 10.1093/jxb/ery059 29474730

[pld3571-bib-0014] Chochois, V. , Vogel, J. P. , Rebetzke, G. J. , & Watt, M. (2015). Variation in adult plant phenotypes and partitioning among seed and stem‐borne roots across *Brachypodium distachyon* accessions to exploit in breeding cereals for well‐watered and drought environments. Plant Physiology, 168, 953–967. 10.1104/pp.15.00095 25975834 PMC4741322

[pld3571-bib-0079] Condon, A. G. (2020). Drying times: Plant traits to improve crop water use efficiency and yield. Journal of Experimental Botany, 71(7), 2239–2252. 10.1093/jxb/eraa002 31912130

[pld3571-bib-0015] Condon, A. G. , Richards, R. A. , Rebetzke, G. J. , & Farquhar, G. D. (2004). Breeding for high water‐use efficiency. Journal of Experimental Botany, 55, 2447–2460. 10.1093/jxb/erh277 15475373

[pld3571-bib-0016] Czedik‐Eysenberg, A. , Seitner, S. , Güldener, U. , Koemeda, S. , Jez, J. , Colombini, M. , & Djamei, A. (2018). The ‘PhenoBox’, a flexible, automated, open‐source plant phenotyping solution. New Phytologist, 219, 808–823. 10.1111/nph.15129 29621393 PMC6485332

[pld3571-bib-0017] Darko, E. , Végh, B. , Khalil, R. , Marček, T. , Szalai, G. , Pál, M. , & Janda, T. (2019). Metabolic responses of wheat seedlings to osmotic stress induced by various osmolytes under iso‐osmotic conditions. PLoS ONE, 14, e0226151. 10.1371/journal.pone.0226151 31856179 PMC6922385

[pld3571-bib-0018] Dodd, A. N. , Kusakina, J. , Hall, A. , Gould, P. D. , & Hanaoka, M. (2014). The circadian regulation of photosynthesis. Photosynthesis Research, 119, 181–190. 10.1007/s11120-013-9811-8 23529849

[pld3571-bib-0019] Ehrenfeld, J. G. , Ravit, B. , & Elgersma, K. (2005). Feedback in the plant‐soil system. Annual Review of Environment and Resources, 30, 75–115. 10.1146/annurev.energy.30.050504.144212

[pld3571-bib-0020] Fageria, N. K. , Baligar, V. C. , & Li, Y. C. (2008). The role of nutrient efficient plants in improving crop yields in the twenty first century. Journal of Plant Nutrition, 31, 1121–1157. 10.1080/01904160802116068

[pld3571-bib-0021] Fahlgren, N. , Feldman, M. , Gehan, M. A. , Wilson, M. S. , Shyu, C. , Bryant, D. W. , Hill, S. T. , McEntee, C. J. , Warnasooriya, S. N. , Kumar, I. , Ficor, T. , Turnipseed, S. , Gilbert, K. B. , Brutnell, T. P. , Carrington, J. C. , Mockler, T. C. , & Baxter, I. (2015). A versatile phenotyping system and analytics platform reveals diverse temporal responses to water availability in Setaria. Molecular Plant, 8, 1520–1535. 10.1016/j.molp.2015.06.005 26099924

[pld3571-bib-0022] Falhof, J. , Pedersen, J. T. , Fuglsang, A. T. , & Palmgren, M. (2016). Plasma membrane H+‐ATPase regulation in the Center of Plant Physiology. Molecular Plant, 9, 323–337. 10.1016/j.molp.2015.11.002 26584714

[pld3571-bib-0023] Farquhar, G. , O'Leary, M. , & Berry, J. (1982). On the relationship between carbon isotope discrimination and the intercellular carbon dioxide concentration in leaves. Functional Plant Biology, 9, 121. 10.1071/PP9820121

[pld3571-bib-0024] Fletcher, A. , Christopher, J. , Hunter, M. , Rebetzke, G. , & Chenu, K. (2018). A low‐cost method to rapidly and accurately screen for transpiration efficiency in wheat. Plant Methods, 14, 77. 10.1186/s13007-018-0339-y 30181766 PMC6116455

[pld3571-bib-0025] Foley, J. A. , DeFries, R. , Asner, G. P. , Barford, C. , Bonan, G. , Carpenter, S. R. , Chapin, F. S. , Coe, M. T. , Daily, G. C. , Gibbs, H. K. , Helkowski, J. H. , Holloway, T. , Howard, E. A. , Kucharik, C. J. , Monfreda, C. , Patz, J. A. , Prentice, I. C. , Ramankutty, N. , & Snyder, P. K. (2005). Global consequences of land use. Science, 309, 570–574. 10.1126/science.1111772 16040698

[pld3571-bib-0026] Ge, T. , Sui, F. , Bai, L. , Tong, C. , & Sun, N. (2012). Effects of water stress on growth, biomass partitioning, and water‐use efficiency in summer maize (*Zea mays* L.) throughout the growth cycle. Acta Physiologiae Plantarum, 34, 1043–1053. 10.1007/s11738-011-0901-y

[pld3571-bib-0027] Gehan, M. A. , Fahlgren, N. , Abbasi, A. , Berry, J. C. , Callen, S. T. , Chavez, L. , Doust, A. N. , Feldman, M. J. , Gilbert, K. B. , Hodge, J. G. , Hoyer, J. S. , Lin, A. , Liu, S. , Lizárraga, C. , Lorence, A. , Miller, M. , Platon, E. , Tessman, M. , & Sax, T. (2017). PlantCV v2: Image analysis software for high‐throughput plant phenotyping. PeerJ, 5, e4088. 10.7717/peerj.4088 29209576 PMC5713628

[pld3571-bib-0028] Granier, C. , & Vile, D. (2014). Phenotyping and beyond: Modelling the relationships between traits. Current Opinion in Plant Biology, 18, 96–102. 10.1016/j.pbi.2014.02.009 24637194

[pld3571-bib-0029] Grossiord, C. , Buckley, T. N. , Cernusak, L. A. , Novick, K. A. , Poulter, B. , Siegwolf, R. T. W. , Sperry, J. S. , & McDowell, N. G. (2020). Plant responses to rising vapor pressure deficit. New Phytologist, 226, 1550–1566. 10.1111/nph.16485 32064613

[pld3571-bib-0030] Großkinsky, D. K. , Svensgaard, J. , Christensen, S. , & Roitsch, T. (2015). Plant phenomics and the need for physiological phenotyping across scales to narrow the genotype‐to‐phenotype knowledge gap. Journal of Experimental Botany, 66, 5429–5440. 10.1093/jxb/erv345 26163702

[pld3571-bib-0031] Grzesiak, M. T. , Grzesiak, S. , & Skoczowski, A. (2006). Changes of leaf water potential and gas exchange during and after drought in triticale and maize genotypes differing in drought tolerance. Photosynthetica, 44, 561–568. 10.1007/s11099-006-0072-z

[pld3571-bib-0032] Henderson, S. , Caemmerer, S. V. , Farquhar, G. D. , Wade, L. , & Hammer, G. (1998). Correlation between carbon isotope discrimination and transpiration efficiency in lines of the C4 species *Sorghum bicolor* in the glasshouse and the field. Functional Plant Biology, 25, 111. 10.1071/PP95033

[pld3571-bib-0033] Hubick, K. , Farquhar, G. , & Shorter, R. (1986). Correlation between water‐use efficiency and carbon isotope discrimination in diverse peanut (Arachis) germplasm. Functional Plant Biology, 13, 803. 10.1071/PP9860803

[pld3571-bib-0034] Kang, S. , Shi, W. , & Zhang, J. (2000). An improved water‐use efficiency for maize grown under regulated deficit irrigation. Field Crops Research, 67, 207–214. 10.1016/S0378-4290(00)00095-2

[pld3571-bib-0035] Kardol, P. , De Deyn, G. B. , Laliberté, E. , Mariotte, P. , & Hawkes, C. V. (2013). Biotic plant–soil feedbacks across temporal scales. Journal of Ecology, 101, 309–315. 10.1111/1365-2745.12046

[pld3571-bib-0036] Kassambara A (2023). ggpubr: ‘ggplot2’ Based Publication Ready Plots. R package version 0.6.0, https://rpkgs.datanovia.com/ggpubr/

[pld3571-bib-0037] Khalifa, M. , & Eltahir, E. A. B. (2023). Assessment of global sorghum production, tolerance, and climate risk. Frontiers in Sustainable Food Systems, 7, 1184373. 10.3389/fsufs.2023.1184373

[pld3571-bib-0038] Lambers, H. , & Oliveira, R. S. (2019). Role in ecosystem and global processes: Ecophysiological controls. In H. Lambers & R. S. Oliveira (Eds.), Plant physiological ecology (pp. 677–698). Springer International Publishing. 10.1007/978-3-030-29639-1_19

[pld3571-bib-0039] Leakey, A. D. B. , Ferguson, J. N. , Pignon, C. P. , Wu, A. , Jin, Z. , Hammer, G. L. , & Lobell, D. B. (2019). Water use efficiency as a constraint and target for improving the resilience and productivity of C3 and C4 crops. Annual Review of Plant Biology, 70, 781–808. 10.1146/annurev-arplant-042817-040305 31035829

[pld3571-bib-0040] Lenth, R. V. (2016). Least‐squares means: The R package lsmeans. Journal of Statistical Software, 69, 1–33.

[pld3571-bib-0041] Li, Z. , Guo, R. , Li, M. , Chen, Y. , & Li, G. (2020). A review of computer vision technologies for plant phenotyping. Computers and Electronics in Agriculture, 176, 105672. 10.1016/j.compag.2020.105672

[pld3571-bib-0042] Liu, D. , Paul, A.‐L. , Morgan, K. T. , & Liu, G. (2022). Effects of oxygen fertilization on damage reduction in flooded snap bean (*Phaseolus vulgaris* L.). Scientific Reports, 12, 4282. 10.1038/s41598-022-08165-5 35277544 PMC8917216

[pld3571-bib-0043] Luo, H. , Zhao, W. , Wang, Y. , Xia, Y. , Wu, X. , Zhang, L. , Tang, B. , Zhu, J. , Fang, L. , du, Z. , Bekele, W. A. , Tai, S. , Jordan, D. R. , Godwin, I. D. , Snowdon, R. J. , Mace, E. S. , Luo, J. , & Jing, H. C. (2016). SorGSD: A sorghum genome SNP database. Biotechnology for Biofuels, 9, 6. 10.1186/s13068-015-0415-8 26744602 PMC4704391

[pld3571-bib-0044] Marschner, H. , Römheld, V. , Horst, W. J. , & Martin, P. (1986). Root‐induced changes in the rhizosphere: Importance for the mineral nutrition of plants. Zeitschrift für Pflanzenernährung Und Bodenkunde, 149, 441–456. 10.1002/jpln.19861490408

[pld3571-bib-0077] Mastrorilli, M. , Katerji, N. , & Rana, G. (1999). Productivity and water use efficiency of sweet sorghum as affected by soil water deficit occurring at different vegetative growth stages. European Journal of Agronomy, 11(3–4), 207–215. 10.1016/s1161-0301(99)00032-5

[pld3571-bib-0045] Medrano, H. , Tomás, M. , Martorell, S. , Flexas, J. , Hernández, E. , Rosselló, J. , Pou, A. , Escalona, J.‐M. , & Bota, J. (2015). From leaf to whole‐plant water use efficiency (WUE) in complex canopies: Limitations of leaf WUE as a selection target. The Crop Journal, 3, 220–228. 10.1016/j.cj.2015.04.002

[pld3571-bib-0046] Menz, M. A. , Klein, R. R. , Unruh, N. C. , Rooney, W. L. , Klein, P. E. , & Mullet, J. E. (2004). Genetic diversity of public Inbreds of sorghum determined by mapped AFLP and SSR markers. Crop Science, 44, 1236–1244. 10.2135/cropsci2004.1236

[pld3571-bib-0047] Miller, F. R. (1984). Registration of RTx430 sorghum parental line. Crop Science, 24, 1224–1224. 10.2135/cropsci1984.0011183X002400060074x

[pld3571-bib-0048] Mortlock, M. Y. , & Hammer, G. L. (2000). Genotype and water limitation effects on transpiration efficiency in sorghum. Journal of Crop Production, 2, 265–286. 10.1300/J144v02n02_11

[pld3571-bib-0049] Murray, F. W. (1967). On the computation of saturation vapor pressure. Journal of Applied Meteorology and Climatology, 6, 203–204. 10.1175/1520-0450(1967)006<0203:OTCOSV>2.0.CO;2

[pld3571-bib-0050] Narsai, R. , Rocha, M. , Geigenberger, P. , Whelan, J. , & van Dongen, J. T. (2011). Comparative analysis between plant species of transcriptional and metabolic responses to hypoxia. New Phytologist, 190, 472–487. 10.1111/j.1469-8137.2010.03589.x 21244431

[pld3571-bib-0051] Paine, C. E. T. , Marthews, T. R. , Vogt, D. R. , Purves, D. , Rees, M. , Hector, A. , & Turnbull, L. A. (2012). How to fit nonlinear plant growth models and calculate growth rates: An update for ecologists. Methods in Ecology and Evolution, 3, 245–256. 10.1111/j.2041-210X.2011.00155.x

[pld3571-bib-0052] Panter, S. N. , & Jones, D. A. (2002). Age‐related resistance to plant pathogens. Advances in Botanical Research. Academic Press, 38, 251–280. 10.1016/S0065-2296(02)38032-7

[pld3571-bib-0053] Pennisi, B. V. , & Thomas, P. A. (2005). Essential pH management in greenhouse crops. University of Georgia, Bulletin 1256.

[pld3571-bib-0054] Peters, W. , van der Velde, I. R. , van Schaik, E. , Miller, J. B. , Ciais, P. , Duarte, H. F. , van der Laan‐Luijkx, I. T. , van der Molen, M. K. , Scholze, M. , Schaefer, K. , Vidale, P. L. , Verhoef, A. , Wårlind, D. , Zhu, D. , Tans, P. P. , Vaughn, B. , & White, J. W. C. (2018). Increased water‐use efficiency and reduced CO2 uptake by plants during droughts at a continental scale. Nature Geoscience, 11, 744–748. 10.1038/s41561-018-0212-7 PMC6179136 30319710

[pld3571-bib-0055] R Core Team . (2021). R: A language and environment for statistical computing. R Foundation for Statistical Computing. https://www.R-project.org/

[pld3571-bib-0056] Rascher, U. , Blossfeld, S. , Fiorani, F. , Jahnke, S. , Jansen, M. , Kuhn, A. J. , Matsubara, S. , Märtin, L. L. A. , Merchant, A. , Metzner, R. , Müller‐Linow, M. , Nagel, K. A. , Pieruschka, R. , Pinto, F. , Schreiber, C. M. , Temperton, V. M. , Thorpe, M. R. , Dusschoten, D. V. , van Volkenburgh, E. , … Schurr, U. (2011). Non‐invasive approaches for phenotyping of enhanced performance traits in bean. Functional Plant Biology, 38, 968–983. 10.1071/FP11164 32480955

[pld3571-bib-0057] Reed, D. W. (Ed.). (1996). A grower's guide to water, media, and nutrition for greenhouse crops. Ball Pub.

[pld3571-bib-0058] Rellán‐Álvarez, R. , Lobet, G. , Lindner, H. , Pradier, P. L. , Sebastian, J. , Yee, M. C. , Geng, Y. , Trontin, C. , LaRue, T. , Schrager‐Lavelle, A. , Haney, C. H. , Nieu, R. , Maloof, J. , Vogel, J. P. , & Dinneny, J. R. (2015). GLO‐roots: An imaging platform enabling multidimensional characterization of soil‐grown root systems. (MJ Harrison, Ed.). eLife, 4, e07597. 26287479 10.7554/eLife.07597 PMC4589753

[pld3571-bib-0059] Resnik, M. E. (1970). Effect of mannitol and polyethylene glycol on phosphorus uptake by maize plants. Annals of Botany, 34, 497–504. 10.1093/oxfordjournals.aob.a084385

[pld3571-bib-0060] Ryan, P. R. , Dessaux, Y. , Thomashow, L. S. , & Weller, D. M. (2009). Rhizosphere engineering and management for sustainable agriculture. Plant and Soil, 321, 363–383. 10.1007/s11104-009-0001-6

[pld3571-bib-0061] Shakoor, N. , Lee, S. , & Mockler, T. C. (2017). High throughput phenotyping to accelerate crop breeding and monitoring of diseases in the field. Current Opinion in Plant Biology, 38, 184–192. 10.1016/j.pbi.2017.05.006 28738313

[pld3571-bib-0062] Shen, J. , Li, C. , Mi, G. , Li, L. , Yuan, L. , Jiang, R. , & Zhang, F. (2013). Maximizing root/rhizosphere efficiency to improve crop productivity and nutrient use efficiency in intensive agriculture of China. Journal of Experimental Botany, 64, 1181–1192. 10.1093/jxb/ers342 23255279

[pld3571-bib-0063] Sinclair, T. R. (2012). Is transpiration efficiency a viable plant trait in breeding for crop improvement? Functional Plant Biology, 39, 359–365. 10.1071/FP11198 32480788

[pld3571-bib-0064] Sinclair, T. R. , & Rufty, T. W. (2012). Nitrogen and water resources commonly limit crop yield increases, not necessarily plant genetics. Global Food Security, 1, 94–98. 10.1016/j.gfs.2012.07.001

[pld3571-bib-0065] Sivakumar, J. , Prashanth, J. E. P. , Rajesh, N. , Reddy, S. M. , & Pinjari, O. B. (2020). Principal component analysis approach for comprehensive screening of salt stress‐tolerant tomato germplasm at the seedling stage. Journal of Biosciences, 45, 141. 10.1007/s12038-020-00111-9 33361632

[pld3571-bib-0066] Slama, I. , Ghnaya, T. , Hessini, K. , Messedi, D. , Savouré, A. , & Abdelly, C. (2007). Comparative study of the effects of mannitol and PEG osmotic stress on growth and solute accumulation in Sesuvium portulacastrum. Environmental and Experimental Botany, 61, 10–17. 10.1016/j.envexpbot.2007.02.004

[pld3571-bib-0067] Sugiyama, A. , Bakker, M. G. , Badri, D. V. , Manter, D. K. , & Vivanco, J. M. (2013). Relationships between Arabidopsis genotype‐specific biomass accumulation and associated soil microbial communities. Botany, 91, 123–126. 10.1139/cjb-2012-0217

[pld3571-bib-0068] Vadez, V. , Kholova, J. , Medina, S. , Kakkera, A. , & Anderberg, H. (2014). Transpiration efficiency: New insights into an old story. Journal of Experimental Botany, 65, 6141–6153. 10.1093/jxb/eru040 24600020

[pld3571-bib-0069] Williams, B. A. , Grantham, H. S. , Watson, J. E. M. , Alvarez, S. J. , Simmonds, J. S. , Rogéliz, C. A. , da Silva, M. , Forero‐Medina, G. , Etter, A. , Nogales, J. , Walschburger, T. , Hyman, G. , & Beyer, H. L. (2020). Minimising the loss of biodiversity and ecosystem services in an intact landscape under risk of rapid agricultural development. Environmental Research Letters, 15, 014001. 10.1088/1748-9326/ab5ff7

[pld3571-bib-0070] Xie, T. , & Su, P. (2012). Canopy and leaf photosynthetic characteristics and water use efficiency of sweet sorghum under drought stress. Russian Journal of Plant Physiology, 59, 224–234. 10.1134/S1021443712020197

[pld3571-bib-0071] Xin, Z. , Aiken, R. , & Burke, J. (2009). Genetic diversity of transpiration efficiency in sorghum. Field Crops Research, 111, 74–80. 10.1016/j.fcr.2008.10.010

[pld3571-bib-0072] Xin, Z. , Franks, C. , Payton, P. , & Burke, J. J. (2008). A simple method to determine transpiration efficiency in sorghum. Field Crops Research, 107, 180–183. 10.1016/j.fcr.2008.02.006

[pld3571-bib-0073] Xu, W. , Cui, K. , Xu, A. , Nie, L. , Huang, J. , & Peng, S. (2015). Drought stress condition increases root to shoot ratio via alteration of carbohydrate partitioning and enzymatic activity in rice seedlings. Acta Physiologiae Plantarum, 37, 9. 10.1007/s11738-014-1760-0

[pld3571-bib-0074] Zeng, D. , Li, M. , Jiang, N. , Ju, Y. , Schreiber, H. , Chambers, E. , Letscher, D. , Ju, T. , & Topp, C. N. (2021). TopoRoot: A method for computing hierarchy and fine‐grained traits of maize roots from 3D imaging. Plant Methods, 17, 127. 10.1186/s13007-021-00829-z 34903248 PMC8667396

[pld3571-bib-0075] Zhao, X. , Wang, W. , Zhang, F. , Deng, J. , Li, Z. , & Fu, B. (2014). Comparative metabolite profiling of two rice genotypes with contrasting salt stress tolerance at the seedling stage. PLoS ONE, 9, e108020. 10.1371/journal.pone.0108020 25265195 PMC4179258

