# Supplementary material for: Non‐destructive, whole‐plant phenotyping reveals dynamic changes in water use efficiency, photosynthesis, and rhizosphere acidification of sorghum accessions under osmotic stress
Source: Plant Direct. 2024 Mar 7;8(3):e571. doi: 10.1002/pld3.571 (PMC10918709; doi:10.1002/pld3.571)
Supplement: Supplementary file 1 — Figure S1: Images of Botanicare fittings used in the ebb and flow system. (A) threaded bulkhead fitting which is inserted into both holes that are drilled into the upper reservoir; (B) threaded plastic debris screen; (C) threaded height extender; (D) debris screen screwed onto bulkhead fitting for water inflow into the upper reservoir; (E) debris screen screwed onto two height extenders for water flowing out of the upper reservoir and back down into the lower reservoir. Images of Botanicare fittings are from https://www.hawthornegc.com. Figure S2: (A) Biomass accumulation, (B) water use, and (C) WUE of plants grown in soil (brown) or hydroponically (blue). Error bars represent the standard deviation (n = 8–22; N = 3–4). Letters represent significantly different groups as determined by two‐way ANOVA followed by Tukey's HSD test (alpha = 0.05). n, number of biological replicates per genotype and condition; N, number of independent experiments. Figure S3: Water loss due to evaporation in closed hydroponic tubes. Black dots represent average cumulative evaporative water loss from closed tubes from which plant stems and roots were removed. Error bars represent the standard deviation (n = 7–23; N = 2–3). n, number of biological replicates per genotype and condition; N, number of independent experiments. Figure S4: (A) Cumulative biomass accumulation as a function of cumulative water use, (B) WUE as a function of total (8‐day) biomass accumulation, and (C) WUE as a function of total (8‐day) water use in control (green) or mannitol‐treated (orange) conditions (n = 12–18 per genotype, condition, and day; N = 4). Thick solid lines represent the treatment‐level linear regression bounded by the 95% confidence interval. Thin lines in A represent linear regressions of individual samples. β values represent the slope (coefficient) of the linear regression. r2 values represent the square of the Pearson correlation coefficient of the linear regression. Asterisks indicate whether the [file PLD3-8-e571-s001.docx]

**Supplemental Figures:**

| **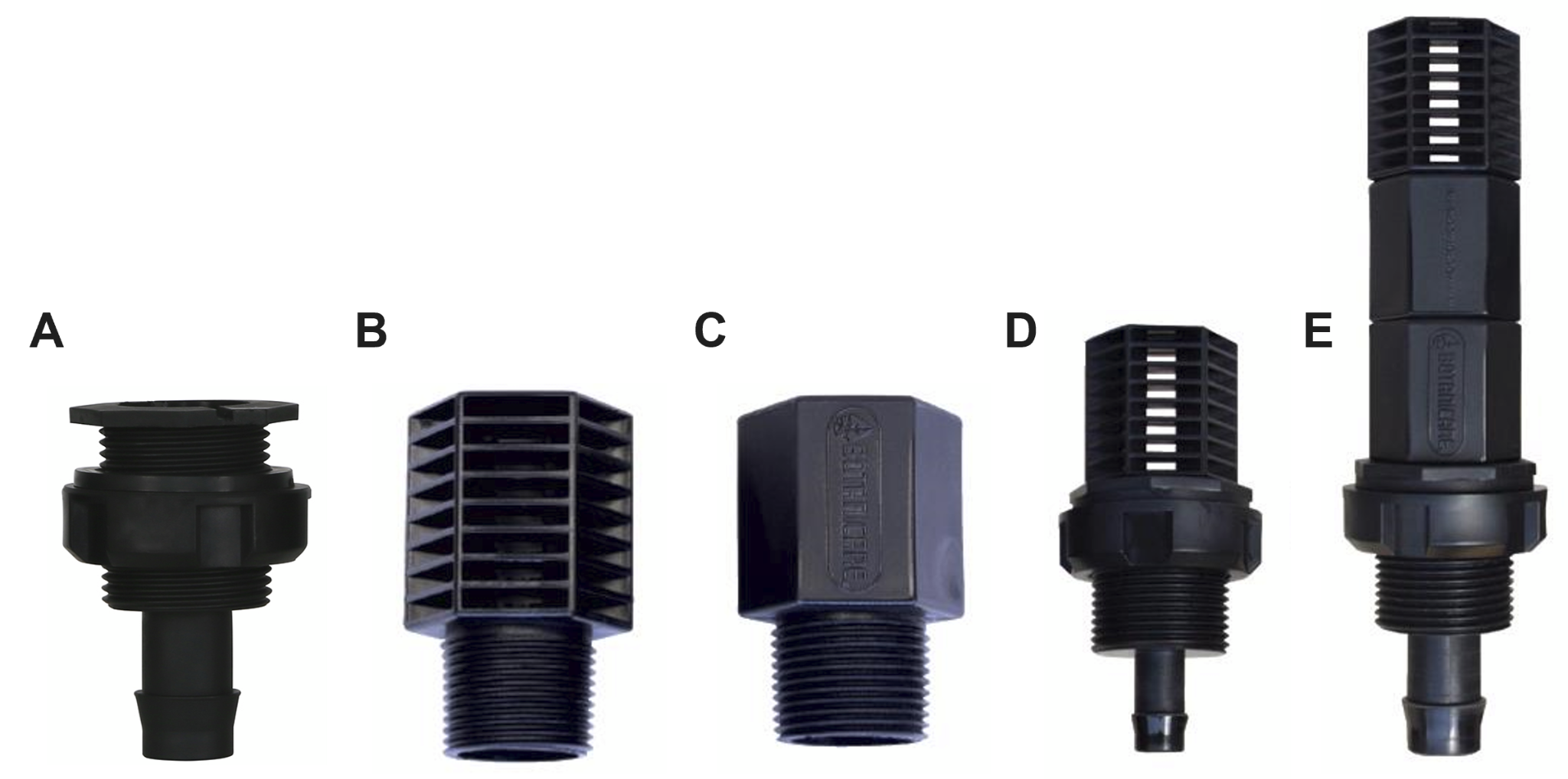** |
| --- |
| **Figure S1:** Images of Botanicare fittings used in the ebb and flow system. (A) threaded bulkhead fitting which is inserted into both holes that are drilled into the upper reservoir; (B) threaded plastic debris screen; (C) threaded height extender; (D) debris screen screwed onto bulkhead fitting for water inflow into the upper reservoir; (E) debris screen screwed onto two height extenders for water flowing out of the upper reservoir and back down into the lower reservoir. Images of Botanicare fittings are from https://www.hawthornegc.com. |

| **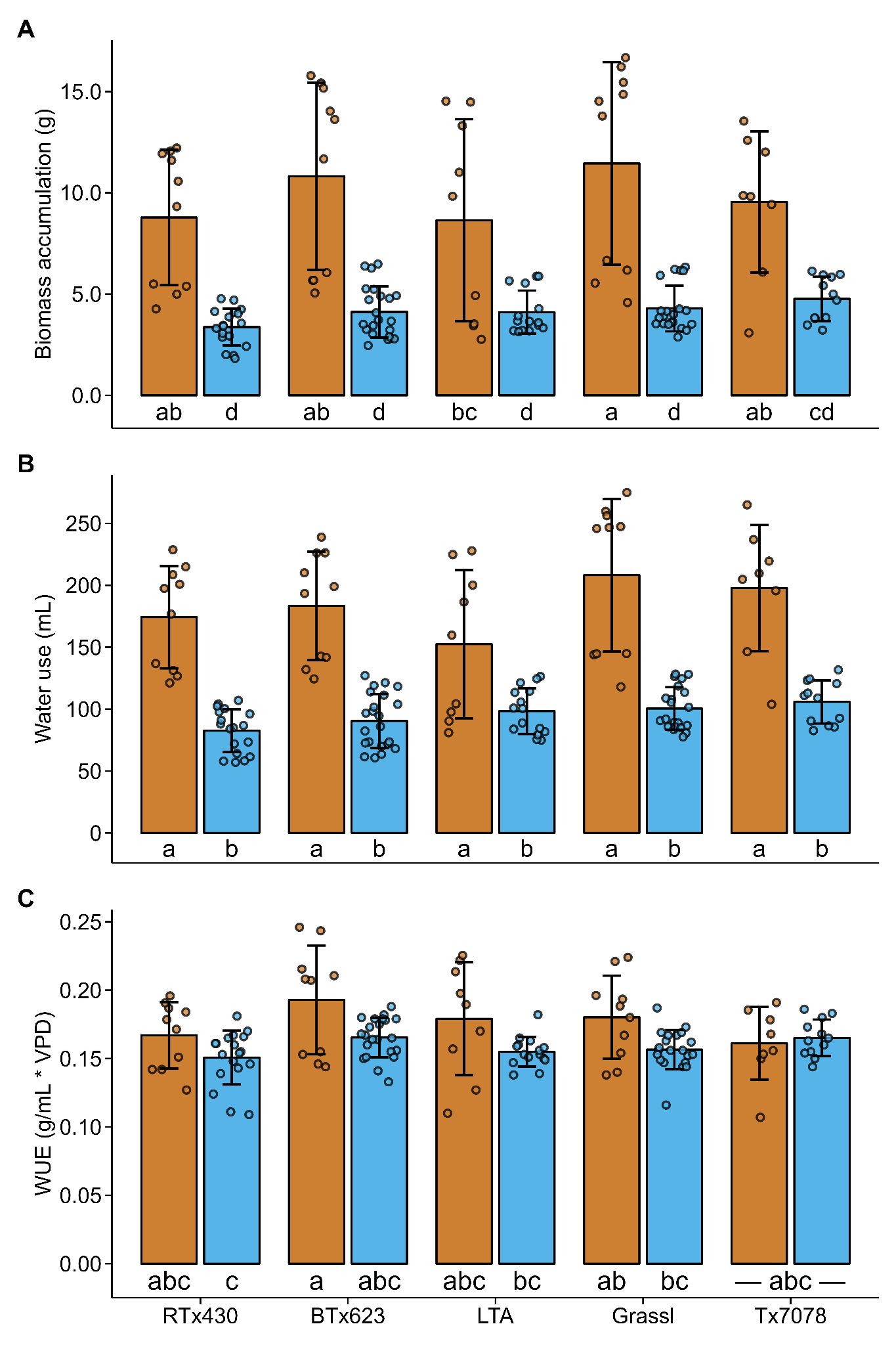** |
| --- |
| **Figure S2:** (A) Biomass accumulation, (B) water use, and (C) WUE of plants grown in soil (brown) or hydroponically (blue). Error bars represent the standard deviation (n = 8-22; N = 3-4). Letters represent significantly different groups as determined by two-way ANOVA followed by Tukey’s HSD test (alpha = 0.05). n, number of biological replicates per genotype and condition; N, number of independent experiments. |

| 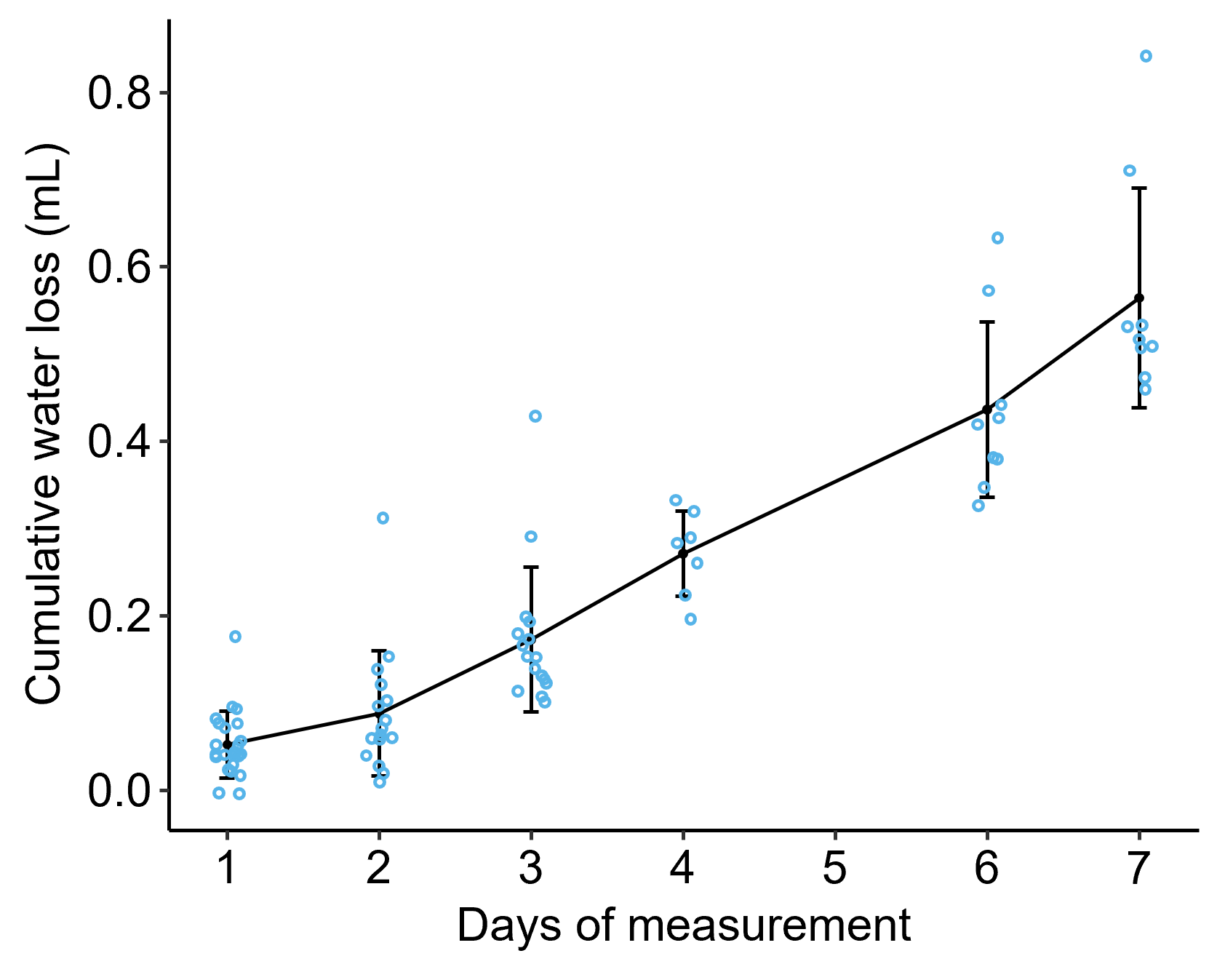 |
| --- |
| **Figure S3:** Water loss due to evaporation in closed hydroponic tubes. Black dots represent average cumulative evaporative water loss from closed tubes from which plant stems and roots were removed. Error bars represent the standard deviation (n = 7-23; N = 2-3). n, number of biological replicates per genotype and condition; N, number of independent experiments. |

| 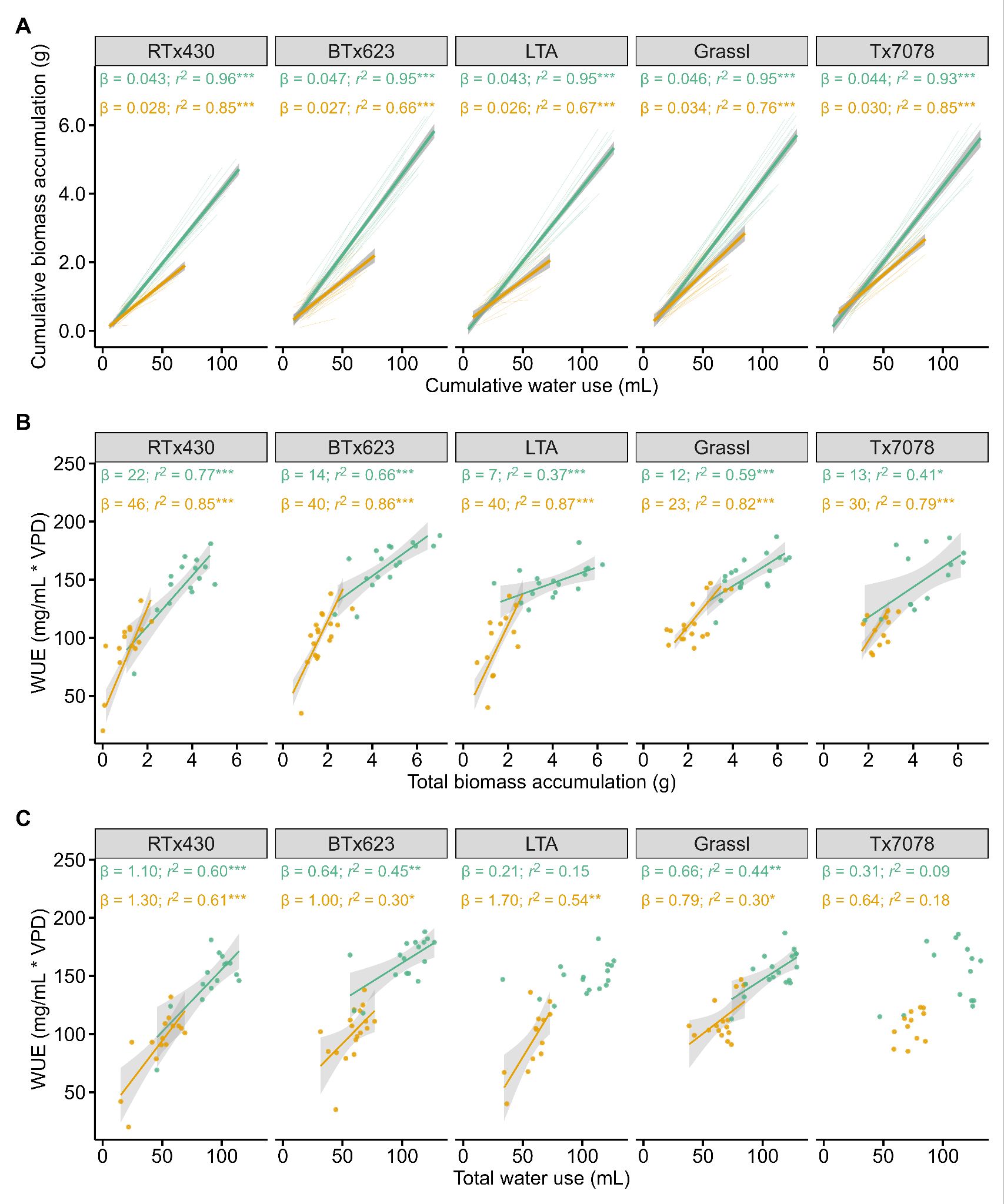 |
| --- |
| **Figure S4:** (**A**) Cumulative biomass accumulation as a function of cumulative water use, (**B**) WUE as a function of total (8-day) biomass accumulation, and **(C**) WUE as a function of total (8-day) water use in control (green) or mannitol-treated (orange) conditions (n = 12-18 per genotype, condition, and day; N = 4). Thick solid lines represent the treatment-level linear regression bounded by the 95% confidence interval. Thin lines in A represent linear regressions of individual samples. β values represent the slope (coefficient) of the linear regression. r^2^ values represent the square of the Pearson correlation coefficient of the linear regression. Asterisks indicate whether the linear relationship is statistically significant: * (p-value < 0.05), ** (p-value < 0.01), *** (p-value < 0.001). Absence of a regression line indicates a non-significant linear relationship (p-value > 0.05). n, number of biological replicates per genotype and condition; N, number of independent experiments. |

**Supplemental File 1:** Initial pH, final pH, and delta-pH for all genotypes, conditions, and days of measurement.
